# How much protein can the body use in a single meal for muscle-building? Implications for daily protein distribution

**DOI:** 10.1186/s12970-018-0215-1

**Published:** 2018-02-27

**Authors:** Brad Jon Schoenfeld, Alan Albert Aragon

**Affiliations:** 1 CUNY Lehman College, Department of Health Sciences, 250 Bedford Park Blvd West, Bronx, NY 10468 USA; 2 California State University, 18111 Nordhoff St, Northridge, CA 91330 USA

**Keywords:** Protein feeding pattern, Amino acid oxidation, Protein intake, Protein metabolism, Lean tissue mass

## Abstract

Controversy exists about the maximum amount of protein that can be utilized for lean tissue-building purposes in a single meal for those involved in regimented resistance training. It has been proposed that muscle protein synthesis is maximized in young adults with an intake of ~ 20–25 g of a high-quality protein; anything above this amount is believed to be oxidized for energy or transaminated to form urea and other organic acids. However, these findings are specific to the provision of fast-digesting proteins without the addition of other macronutrients. Consumption of slower-acting protein sources, particularly when consumed in combination with other macronutrients, would delay absorption and thus conceivably enhance the utilization of the constituent amino acids. The purpose of this paper was twofold: 1) to objectively review the literature in an effort to determine an upper anabolic threshold for per-meal protein intake; 2) draw relevant conclusions based on the current data so as to elucidate guidelines for per-meal daily protein distribution to optimize lean tissue accretion. Both acute and long-term studies on the topic were evaluated and their findings placed into context with respect to per-meal utilization of protein and the associated implications to distribution of protein feedings across the course of a day. The preponderance of data indicate that while consumption of higher protein doses (> 20 g) results in greater AA oxidation, this is not the fate for all the additional ingested AAs as some are utilized for tissue-building purposes. Based on the current evidence, we conclude that to maximize anabolism one should consume protein at a target intake of 0.4 g/kg/meal across a minimum of four meals in order to reach a *minimum* of 1.6 g/kg/day. Using the upper daily intake of 2.2 g/kg/day reported in the literature spread out over the same four meals would necessitate a maximum of 0.55 g/kg/meal.

## Background

Controversy exists about the maximum amount of protein that can be utilized for lean tissue-building purposes in a single meal for those involved in regimented resistance training. A long-held misperception in the lay public is that there is a limit to how much protein can be absorbed by the body. From a nutritional standpoint, the term “absorption” describes the passage of nutrients from the gut into systemic circulation. Based on this definition, the amount of protein that can be absorbed is virtually unlimited. Following digestion of a protein source, the constituent amino acids (AA) are transported through the enterocytes at the intestinal wall, enter the hepatic portal circulation, and the AA that are not utilized directly by the liver, then enter the bloodstream, after which almost all the AA ingested become available for use by tissues. While absorption is not a limiting factor with respect to whole proteins, there may be issues with consumption of individual free-form AA in this regard. Specifically, evidence shows the potential for competition at the intestinal wall, with AA that are present in the highest concentrations absorbed at the expense of those that are less concentrated [1].

It has been proposed that muscle protein synthesis (MPS) is maximized in young adults with an intake of ~ 20–25 g of a high-quality protein, consistent with the “muscle full” concept; anything above this amount is believed to be oxidized for energy or transaminated to form alternative bodily compounds [2]. The purpose of this paper is twofold: 1) to objectively review the literature in an effort to determine an upper anabolic threshold for per-meal protein intake; 2) draw relevant conclusions based on the current data so as to elucidate guidelines for per-meal daily protein distribution to optimize lean tissue accretion.

### Speed of digestion/absorption on muscle anabolism

In a study often cited as support for the hypothesis that MPS is maximized at a protein dose of ~ 20–25 g, Areta et al. [3] provided differing amounts of protein to resistance-trained subjects over a 12-h recovery period following performance of a multi-set, moderate repetition leg-extension exercise protocol. A total of 80 g of whey protein was ingested in one of the following three conditions: 8 servings of 10 g every 1.5 h; 4 servings of 20 g every 3 h; or 2 servings of 40 g every 6 h. Results showed that MPS was greatest in those who consumed 4 servings of 20 g of protein, suggesting no additional benefit, and actually a lower rise in MPS when consuming the higher dosage (40 g) under the conditions imposed in the study. These results extended similar findings by Moore et al. [4] on whole-body nitrogen turnover.

Although the findings of Areta et al. [3] provide interesting insight into the dose-related effects of protein intake on muscle development, it is important to note that a number of factors influence dietary protein metabolism including the composition of the given protein source, the composition of the meal, the amount of protein ingested, and the specifics of the exercise routine [5]. In addition, individual variables such as age, training status, and the amount of lean body mass also impact muscle-building outcomes. A major limitation in the study by Areta et al. [3] is that total protein intake over the 12-h study period was only 80 g, corresponding to less than 1 g/kg of body mass. This is far below the amount necessary to maximize muscle protein balance in resistance-trained individuals who served as participants in the study [6, 7]. Furthermore, the ecological validity of this work is limited since habitual protein intakes of individuals focused on muscle gain or retention habitually consume approximately 2–4 times this amount per day [8, 9].

It also should be noted that subjects in Areta et al. [3] ingested nothing but whey protein throughout the post-exercise period. Whey is a “fast-acting” protein; its absorption rate has been estimated at ~ 10 g per hour [5]. At this rate, it would take just 2 h to fully absorb a 20-g dose of whey. While the rapid availability of AA will tend to spike MPS, earlier research examining whole body protein kinetics showed that concomitant oxidation of some of the AA may result in a lower net protein balance when compared to a protein source that is absorbed at a slower rate [10]. For example, cooked egg protein has an absorption rate of ~ 3 g per hour [5], meaning complete absorption of an omelet containing the same 20 g of protein would take approximately 7 h, which may help attenuate oxidation of AA and thus promote greater whole-body net positive protein balance. An important caveat is that these findings are specific to whole body protein balance; the extent to which this reflects skeletal muscle protein balance remains unclear.

Although some studies have shown similar effects of fast and slow proteins on net muscle protein balance [11] and fractional synthetic rate [12–14], other studies have demonstrated a greater anabolic effect of whey compared to more slowly digested sources both at rest [15, 16], and after resistance exercise [16, 17]. However, the majority of these findings were during shorter testing periods (4 h or less), whereas longer testing periods (5 h or more) tend to show no differences between whey and casein on MPS or nitrogen balance [18]. Furthermore, most studies showing greater anabolism with whey used a relatively small dose of protein (≤20 g) [15–17]; it remains unclear whether higher doses would result in greater oxidation of fast vs slow-acting protein sources.

Compounding these equivocal findings, research examining the fate of intrinsically labeled whey and casein consumed within milk found a greater incorporation of casein into skeletal muscle [19]. The latter finding should be viewed with the caveat that although protein turnover in the leg is assumed to be mostly reflective of skeletal muscle, it is also possible that non-muscle tissues might also contribute. Interestingly, the presence versus absence of milk fat coingested with micellar casein did not delay the rate of protein-derived circulating amino acid availability or myofibrillar protein synthesis [20]. Furthermore, the coingestion of carbohydrate with casein delayed digestion and absorption, but still did not impact muscle protein accretion compared to a protein-only condition [21]. The implication is that accompanying macronutrients’ potential to alter digestion rates does not necessarily translate to alterations in the anabolic effect of the protein feeding – at least in the case of slow-digesting protein such as casein. More fat and/or carbohydrate coingestion comparisons need to be made with other proteins, subject profiles, and relative proximity to training before drawing definitive conclusions.

### Higher acute ‘anabolic ceiling’ than previously thought?

More recently, Macnaughton et al. [22] employed a randomized, double-blind, within-subject design whereby resistance-trained men participated in two trials separated by ~ 2 weeks. During one trial subjects received 20 g of whey protein immediately after performing a total body resistance training bout; during the other trial the same protocol was instituted but subjects received a 40-g whey bolus following training. Results showed that the myofibrillar fractional synthetic rate was ~ 20% higher from consumption of the 40 g compared to the 20 g condition. The researchers speculated that the large amount of muscle mass activated from the total body RT bout necessitated a greater demand for AA that was met by a higher exogenous protein consumption. It should be noted that findings by McNaughton et al. [22] are somewhat in contrast to previous work by Moore et al. showing no statistically significant differences in MPS between provision of a 20 g and 40 g dose of whey in young men following a leg extension bout, although the higher dose produced an 11% greater absolute increase [23]. Whether differences between intakes higher than ~ 20 g per feeding are practically meaningful remain speculative, and likely depend on the goals of the individual.

Given that muscular development is a function of the dynamic balance between MPS and muscle protein breakdown (MPB), both of these variables must be considered in any discussion on dietary protein dosage. Kim et al. [24] endeavored to investigate this topic by provision of either 40 or 70 g of beef protein consumed as part of a mixed meal on two distinct occasions separated by a ~ 1 week washout period. Results showed that the higher protein intake promoted a significantly greater whole-body anabolic response, which was primarily attributed to a greater attenuation of protein breakdown. Given that participants ate large, mixed meals as whole foods containing not only protein, but carbohydrates and dietary fats as well, it is logical to speculate that this delayed digestion and absorption of AAs compared to liquid consumption of isolated protein sources. This, in turn, would have caused a slower release of AA into circulation and hence may have contributed to dose-dependent differences in the anabolic response to protein intake. A notable limitation of the study is that measures of protein balance were taken at the whole-body level and thus not muscle-specific. It therefore can be speculated that some if not much of anti-catabolic benefits associated with higher protein intake was from tissues other than muscle, likely the gut. Even so, protein turnover in the gut potentially provides an avenue whereby accumulated amino acids can be released into the systemic circulation to be used for MPS, conceivably enhancing anabolic potential [25]. This hypothesis remains speculative and warrants further investigation. It would be tempting to attribute these marked reductions in proteolysis to higher insulin responses considering the inclusion of a generous amount of carbohydrate in the meals consumed. Although insulin is often considered an anabolic hormone, its primary role in muscle protein balance is related to anti-catabolic effects [26]. However, in the presence of elevated plasma AAs, the effect of insulin elevations on net muscle protein balance plateaus within a modest range of 15–30 mU/L [27, 28]. Given evidence that a 45 g dose of whey protein causes insulin to rise to levels sufficient to maximize net muscle protein balance [29], it would seem that the additional macronutrients consumed in the study by Kim et al. [24] had little bearing on results.

## Longitudinal findings

Although the previously discussed studies offer insight into how much protein the body can utilize in a given feeding, acute anabolic responses are not necessarily associated with long-term muscular gains [30]. The topic can only be answered by assessing the results of longitudinal studies that directly measure changes in lean mass with provision of varying protein dosages, as well as proteins of varying speeds of digestion/absorption.

Wilborn et al. [31], found no difference in lean mass gains after 8 weeks of pre- and post-resistance exercise supplementation with either whey or casein. Similarly, a lack of between-group differences in lean mass gain was found by Fabre et al. [32] when comparing the following whey/casein protein ratios consumed postexercise: 100/0, 50/50, 20/80.

In a 14-day study of elderly women, Arnal et al. [33] demonstrated that providing a majority of daily protein (79%) in a single meal (pulse pattern) resulted in a greater retention of fat-free mass compared to an evenly distributed intake partitioned over four daily meals (spread pattern). A follow-up study by the same lab in young women reported similar effects of pulse versus spread patterns of protein intake [34]. The combined findings of these studies indicate that muscle mass is not negatively affected by consuming the majority of daily protein as a large bolus. However, neither study employed regimented resistance training thereby limiting generalizability to individuals involved in intense exercise programs.

Insights into the effects of protein dosage can also be gleaned from studies on intermittent fasting (IF). Typical IF protocols require intake of daily nutrients, including protein, in a narrow time-frame – usually less than 8 h – followed by a prolonged fast. A recent systematic review concluded that IF has similar effects on fat-free mass compared with continuous eating protocols [35]. However, the studies reviewed in the analysis generally involved suboptimal protein intakes consumed as part of a low-energy diet without a resistance training component, again limiting the ability to extrapolate findings to resistance-trained individuals.

Helping to fill this literature gap is an 8-week trial by Tinsley et al. [36], comparing a time-restricted feeding (TRF) protocol of 20-h fasting/4-h feeding cycles done 4 days per week, with a normal-diet group (ND) in untrained subjects doing resistance training 3 days per week. The TRF group lost body weight via lower energy intake (667 kcal less on fasting vs. non-fasting days), but did not significantly lose lean mass (0.2 kg); ND gained lean mass (2.3 kg), but not to a statistically significant degree, although the magnitude of differences raises the possibility that these findings may be practically meaningful. Perhaps most interestingly, biceps brachii and rectus femoris cross sectional area showed similar increases in both groups despite the 20-h fasting cycles and concentrated feeding cycles in TRF, suggesting that the utilization of protein intake in the ad libitum 4-h feeding cycles was not hampered by an acute ceiling of anabolism. Unfortunately, protein and enrgy were not equated in this study. Subsequently, an 8-week trial by Moro et al. [37] using resistance-trained subjects on a 16-h fasting/8-h TRF cycle found significantly greater fat loss in TRF vs. ND (1.62 vs. 0.31 kg) while lean mass remained unchanged in both groups. These findings further call into question the concern for breaching a certain threshold of protein intake per meal for the goal of muscle retention.

In contrast to the above findings showing neutral-to-positive effects of a temporally concentrated meal intake, Arciero et al. [38] compared 3 diets: 2 high-protein (35% of total energy) diets consisting of 3 (HP3) and 6 meals/day (HP6), and a traditional protein intake (15% of total energy) consumed in 3 meals/day (TD3). During the initial 28-day eucaloric phase, HP3 and HP6 consumed protein at 2.27 & 2.15 g/kg, respectively, while TD3 consumed 0.9 g/kg. HP6 was the only goup that significantly gained lean mass. During the subsequent 28-day eucaloric phase, HP3 and HP6 consumed protein at 1.71 & 1.65 g/kg, respectively, while TD3 consumed 0.75 g/kg. HP6 maintained its lean mass gain, outperforming the other 2 treatments in this respect (HP actually showed a significant loss of lean mass compared to the control). The discrepancy between the latter findings and those in the IF/TRF trials remains to be reconciled. In any case, it is notable that comparisons in this vein specifically geared toward the goal of muscle gain, hypercaloric comparisons in particular, are lacking.

## Conclusions

An important distinction needs to be made between acute meal challenges comparing different protein amounts (including serial feedings in the acute phase following resistance training) and chronic meal feedings comparing different protein distributions through the day, over the course of several weeks or months. Longitudinal studies examining body composition have not consistently corroborated the results of acute studies examining muscle protein flux. Quantifying a maximum amount of protein per meal that can be utilized for muscle anabolism has been a challenging pursuit due to the multitude of variables open for investigation. Perhaps the most comprehensive synthesis of findings in this area has been done by Morton et al. [2], who concluded that 0.4 g/kg/meal would optimally stimulate MPS. This was based on the addition of two standard deviations to their finding that 0.25 g/kg/meal maximally stimulates MPS in young men. In line with this hypothesis, Moore et al. [39] mentioned the caveat that their findings were estimated means for maximizing MPS, and that the dosing ceilings can be as high as ~ 0.60 g/kg for some older men and ~ 0.40 g/kg for some younger men. Importantly, these estimates are based on the sole provision of a rapidly digesting protein source that would conceivably increase potential for oxidation of AA when consumed in larger boluses. It seems logical that a slower-acting protein source, particularly when consumed in combination with other macronutrients, would delay absorption and thus enhance the utilization of the constituent AA. However, the practical implications of this phenomenon remain speculative and questionable [21].

The collective body of evidence indicates that total daily protein intake for the goal of maximizing resistance training-induced gains in muscle mass and strength is approximately 1.6 g/kg, at least in non-dieting (eucaloric or hypercaloric) conditions [6]. However, 1.6 g/kg/day should not be viewed as an ironclad or universal limit beyond which protein intake will be either wasted or used for physiological demands aside from muscle growth. A recent meta-analysis on protein supplementation involving resistance trainees reported an upper 95% confidence interval (CI) of 2.2 g/kg/day [6]. Bandegan et al. [7] also showed an upper CI of 2.2 g/kg/day in a cohort of young male bodybuilders, although the method of assessment (indicator amino acid oxidation technique) used in this study has not received universal acceptance for determining optimal protein requirements. This reinforces the practical need to individualize dietary programming, and remain open to exceeding estimated averages. It is therefore a relatively simple and elegant solution to consume protein at a target intake of 0.4 g/kg/meal across a minimum of four meals in order to reach a minimum of 1.6 g/kg/day – if indeed the primary goal is to build muscle. Using the upper CI daily intake of 2.2 g/kg/day over the same four meals would necessitate a maximum of 0.55 g/kg/meal. This tactic would apply what is currently known to maximize acute anabolic responses as well as chronic anabolic adaptations. While research shows that consumption of higher protein doses (> 20 g) results in greater AA oxidation [40], evidence indicates that this is not the fate for all the additional ingested AAs as some are utilized for tissue-building purposes. Further research is nevertheless needed to quantify a specific upper threshold for per-meal protein intake.
